# Adsorption of Carbon Dioxide on Mono-Layer Thick Oxidized Samarium Films on Ni(100)

**DOI:** 10.3390/nano11082064

**Published:** 2021-08-14

**Authors:** Steinar Raaen

**Affiliations:** Department of Physics, Norwegian University of Science and Technology (NTNU), 7491 Trondheim, Norway; sraaen@ntnu.no

**Keywords:** carbon dioxide, samarium, Ni(100), adsorption, surface nano-structures, TPD, XPS, UPS, carbonate

## Abstract

Studies of adsorption of CO${}_{2}$ on nanoscopic surfaces are relevant for technological applications in heterogeneous catalysis as well as for sorption of this important greenhouse gas. Presently, adsorption of carbon dioxide on pure and oxidized thin samarium layers near mono-layer thickness on Ni(100) has been investigated by photoelectron spectroscopy and temperature programmed desorption. It is observed that very little CO${}_{2}$ adsorb on the metallic sample for exposures in the vacuum regime at room temperature. For the oxidized sample, a large enhancement in CO${}_{2}$ adsorption is observed in the desorption measurements. Indications of carbonate formation on the surface were found by C 1s and O 1s XPS. After annealing of the oxidized samples to 900 K very little CO${}_{2}$ was found to adsorb. Differences in desorption spectra before and after annealing of the oxidized samples are correlated with changes in XPS intensities, and with changes in sample work function which determines the energy difference between molecular orbitals and substrate Fermi level, and thus the probability of charge transfer between adsorbed molecule and substrate.

## 1. Introduction

Increased interest in recent years in adsorption properties of carbon dioxide has been triggered by applications in chemical industry and by the role of CO${}_{2}$ as the most important greenhouse gas. Carbon dioxide tend to adsorb on oxide surfaces but show less affinity to metal surfaces. The presence of a surface may lead to formation of carbonate, ${\mathrm{CO}}_{2}^{\gamma -}$ species, or dissociation [1]. A comprehensive understanding of such phenomena is important for optimizing technological processes. Nano-structured surfaces open up new degrees of freedom that need to be unraveled. Presently, near mono-layer thick samarium layers on Ni(100) are considered. Ni was chosen as substrate due to the catalytic properties.

Rare earth oxide surfaces play an important role in heterogeneous catalysis. Fields of applications include petroleum chemical industry, catalytic combustion, automotive emission control, and purification of industrial waste [2,3,4,5]. The catalytic activity of rare earth oxides stem from low energy spin and charge fluctuations [6]. Rare earths have many interesting properties caused by the electronic structure which contains a partly filled 4f electron shell. Rare earth oxides have been reported to be hydrophobic [7,8,9], which is beneficial for heat transfer mechanisms in industrial applications. Samarium compounds may exhibit mixed valence behavior since the energy difference between trivalent Sm ($4f^{6}$ electron configuration) and divalent Sm ($4f^{5}$ electron configuration) is small so that perturbations by e.g., compound formation may result in a change of valence state or result in valence fluctuations [10]. For example, Sm metal is trivalent in the bulk, whereas the surface is divalent due to the lower coordination number [11,12]. The low work function of the rare earths and the relative high stability of rare earth oxides make such materials interesting for adsorption studies [13].

Activation of carbon dioxide by charge doping of the molecule through transfer from a catalytic active surface has been extensively studied during the last several years. These works include experimental and theoretical investigations of vacuum adsorption of CO${}_{2}$ at low temperatures on metal surfaces [14,15,16,17] as well as experiments at higher pressures [18,19,20,21,22].

Whereas relevant chemical reactions frequently take place at elevated temperatures and pressures, this paper presents a study of adsorption of carbon dioxide on samarium nano-structures on a Ni(100) substrate at ambient temperatures and in ultra high vacuum. The observed enhanced CO${}_{2}$ adsorption on oxidized samples have bearing on the understanding of capture mechanisms as well as chemical reactions involving the important green house gas. The positions of the electron energy levels of filled and empty molecular orbitals of carbon dioxide relative to the substrate Fermi level are argued to be relevant for the adsorption properties of these samarium based structures.

## 2. Materials and Methods

The Ni(100) crystal was cleaned by repeated Ar+ sputtering, flash heating, and oxygen annealing. The quality of the Ni single crystal was verified by measuring a sharp FCC(100) low-energy electron diffraction (LEED) pattern. Sm was deposited from an electron beam heated evaporation source using an Al-oxide crucible. Temperature programmed desorption (TPD) spectra were obtained by using a shielded and differentially pumped Prisma quadrupole mass spectrometer (Pfeiffer).The mass spectrometer was positioned close to the sample surface during measurements to discriminate against spurious desorption from the sample support, and to obtain reproducible intensities that could be compared for different runs. Spectra were obtained from masses 2, 18, 28, 32, and 44 amu (atomic mass unit) simultaneously for all experimental runs. X-ray photoelectron spectrocopy (XPS) and ultra-violet photoelectron spectrocopy (UPS) measurements were recorded using a SES2002 spectrometer (Scienta) in conjunction with a monochromatized Al Kα X-ray source (Scienta) and a UVS300 He discharge lamp (Specs) which provided photons of energy hν=21.2 eV. The sample work function was estimated by subtracting the width of the UPS spectrum from the photon energy using a sample bias of −5 V. Deposited effective amounts of Sm were estimated from XPS intensities. The electron energy spectrometer was calibrated within ±0.1 eV by using the Ag 3d core level and the Fermi level. Sample cleanliness was monitored by XPS.

## 3. Results

Sm was evaporated at room temperature onto the clean Ni(100) crystal at a rate of about 0.4 mono-layer (ML) per minute. The samples were then exposed to 10 L (Langmuir) $O_{2}$ (1L = 1.33 × 10${}^{-4}$ Pa · s). The oxidized samples were subsequently exposed to 1 L CO${}_{2}$ for the TPD measurements. First, the XPS results are presented, then the UPS and work function results are described, and finally the results from thermal desorption are given.

### 3.1. XPS Measurements

**Figure 1:**
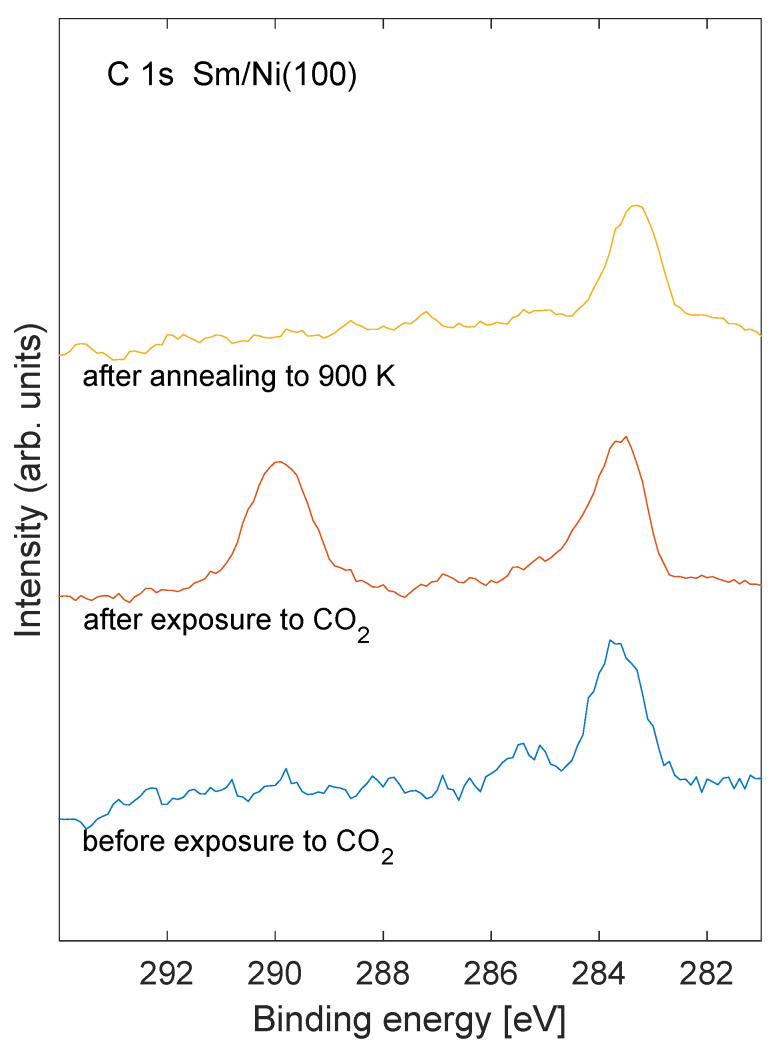
C 1s XPS recorded before exposure to CO${}_{2}$ (**bottom spectrum**), after exposure to CO${}_{2}$ (**middle spectrum**), and after annealing to 900 K (**top spectrum**). The Sm coverage was estimated to 0.8 ML.

**Figure 2:**
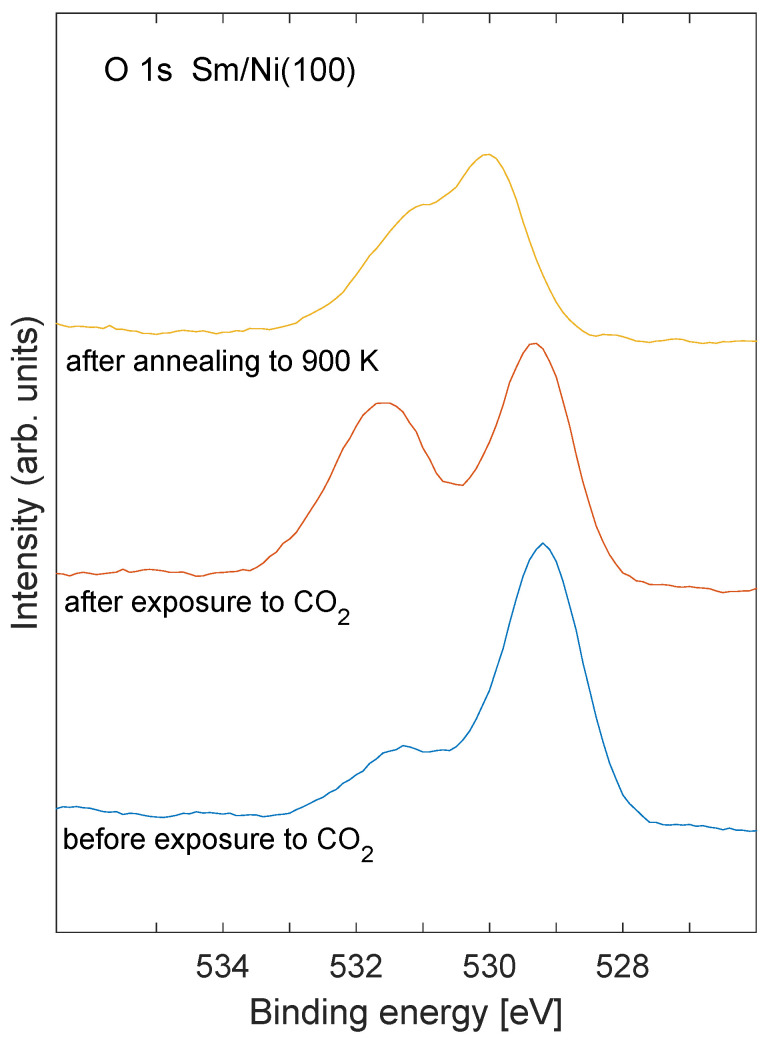
O 1s XPS recorded before exposure to CO${}_{2}$ (**bottom spectrum**), after exposure to CO${}_{2}$ (**middle spectrum**), and after annealing to 900 K (**top spectrum**). The Sm coverage was estimated to 0.8 ML.

Carbon 1s XPS core level spectra are shown in Figure 1 before exposure to CO${}_{2}$ (bottom spectrum), after exposure to CO${}_{2}$ (middle spectrum), and after annealing to 900 K (top spectrum) for Sm/Ni exposed to oxygen. The Sm coverage was estimated to be 0.8 ML for these spectra. Corresponding oxygen 1s core level spectra are shown in Figure 2. The peak that was observed at binding energy near 290 eV for the C 1s core level after CO${}_{2}$ exposure (middle curve in Figure 1) is characteristic of carbonate species at the surface [23]. Similarly, the oxygen 1s emission after CO${}_{2}$ exposure (middle spectrum in Figure 2) shows a peak near binding energy 532 eV which is consistent with carbonate species [24]. The C 1s peak near binding energy 290 eV disappears after annealing the sample ti 900 K (upper spectrum in Figure 1). The intensity of the carbon contamination at binding energy near 283.5 eV (bottom spectrum in Figure 1) was estimated to be about 3% of the Ni signal.

**Figure 3:**
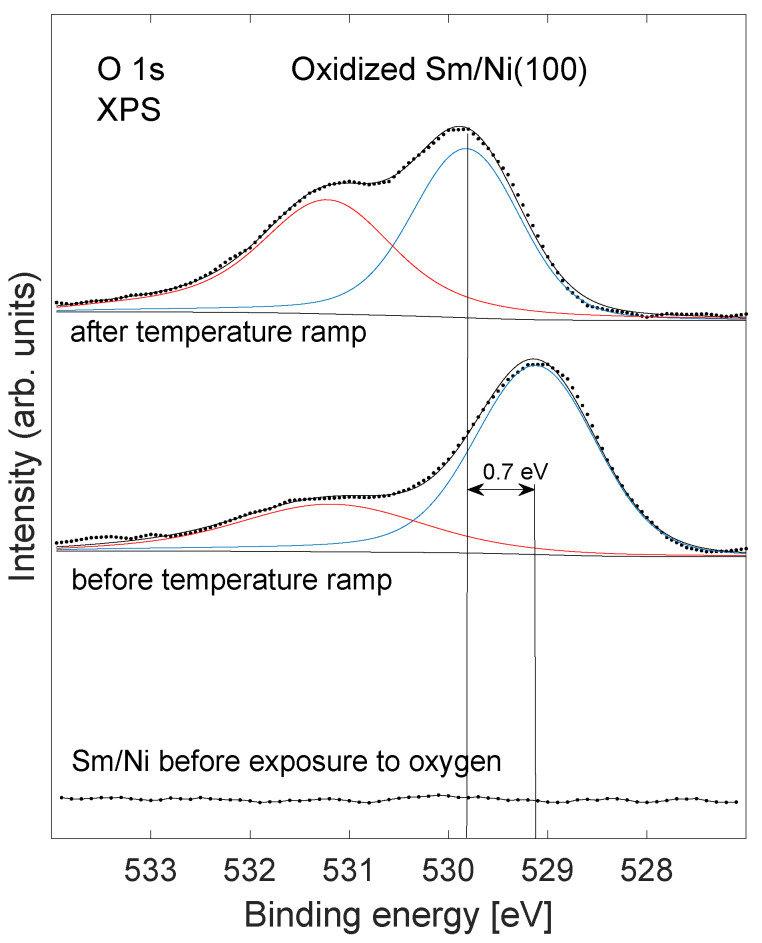
XPS showing the O 1s core level before and after heating to 900 K of an oxidized sample of effective Sm coverage 0.9 ML. A core level shift of the main feature in the spectra of 0.7 eV after heating is indicated in the figure. The bottom curve shows the absence of O 1s emission before exposure to oxygen of the Sm/Ni sample.

Changes in the O 1s core level emission are shown in Figure 3 for a sample of effective Sm coverage 0.9 ML exposed to oxygen as the sample is heated. A core level shift of 0.7 eV was observed for the major component after heating to 900 K. A significant intensity increase of the feature near 531.2 eV was seen subsequent to annealing. The minor peak near 531.2 eV was not shifted upon heating. These findings are addressed in the next section.

**Figure 4:**
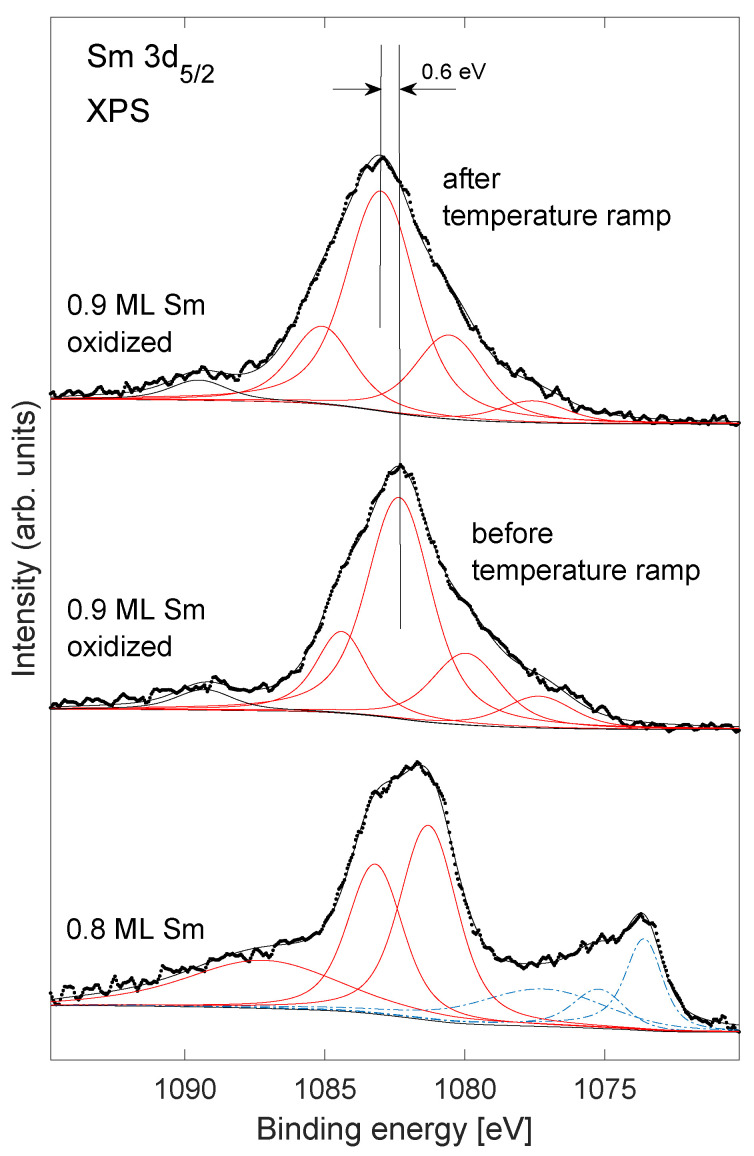
Sm $3d_{5/2}$ XPS for Sm/Ni (**bottom spectrum**), oxidized sample before temperature ramp (**middle spectrum**), and oxidized sample after temperature ramp (**top spectrum**). Effective Sm coverage as indicated in the figure. Solid lines show convolution of Sm${}^{3+}$ components, and dashed lines show the Sm${}^{2+}$ components.

Samarium $3d_{5/2}$ XPS are shown in Figure 4 for Sm/Ni (bottom spectrum) and Sm/Ni exposed to oxygen (two top spectra). The 3d XPS of Sm is made complicated by multiplet interactions between the open 4f shell and the valence electrons [25,26]. Deconvoluted components are shown as solid lines for the Sm${}^{3+}$ components, and as dashed lines for the Sm${}^{2+}$ components (for the metallic spectrum). These components do not represent the true multiplet structure of Sm but merely represent the smallest number of peaks that give a reasonable fit to the data. Only trivalent Sm was observed in the spectra from the samples that were exposed to oxygen. XPS from rare earth oxides is rather complex and the weak components at lower binding energy as compared to the main peak have been identified as due to strong charge transfer of unpaired 4f electrons [27,28]. The main component of the convolution of the spectra of the sample exposed to oxygen is shifted 0.6 eV to higher binding energy after the temperature ramp (see two top spectra in Figure 4). Table 1 lists XPS intensities for Sm 3d and O 1s core levels relative to the intensity of the Ni 2p core level. The intensities have been corrected by using atomic sensitivity factors. It is seen from the table that the Sm intensities show relatively small reductions (≤10%) after annealing to 900 K. For the lowest Sm coverage, 0.4 and 0.9 ML the oxygen intensity decreases substantially. For the higher Sm coverage, 1.9 ML, the relative Sm and O core level intensities showed relatively small increases (<10%) after annealing.

### 3.2. UPS and Work Function Determination

**Figure 5:**
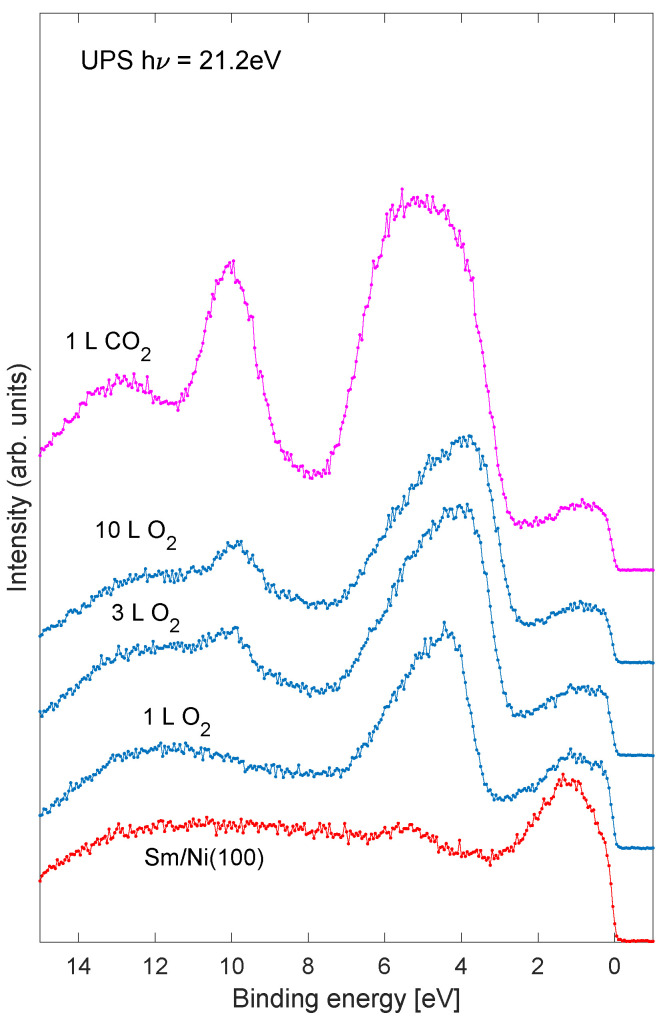
UPS of the valence band region of the Sm/Ni(100) sample. The effective Sm coverage was 0.8 ML.The bottom curve shows the clean Sm/Ni sample. The three curves in the middle show increasing exposure to oxygen, and the top spectrum shows exposure to CO${}_{2}$ on the oxidized sample.

UPS of the valence band region of the Sm/Ni system is shown in Figure 5. The bottom spectrum corresponds to pure Sm/Ni. As the sample is exposed to oxygen the O 2s emission near 5 eV binding energy grows. A feature near 10 eV emerges for the two largest oxygen exposures (middle curves). Upon exposure to carbon dioxide the 10 eV feature increases significantly. This peak is identified as C 2s, possibly originating from carbonate species, as indicated by the C 1s spectra in Figure 1 [29,30].

The work functions of the samples were determined by UPS. Work function changes are shown in Table 2 for Sm/Ni(100), Sm/Ni(100) exposed to oxygen, and CO${}_{2}$ adsorbed on oxidized Sm/Ni(100). The effective Sm coverage was estimated to be near 0.8 ML. The work function for clean Ni(100) was measured to be 5.2 eV. A reduction of 2.3 eV was seen after Sm deposition. An increase in the work function by 0.9 eV was observed after an oxygen exposure of 10 L of the Sm/Ni sample. When the oxidized Sm/Ni sample was exposed to a CO${}_{2}$ dose of 1 L, the work function increased by 0.6 eV. After temperature ramp to 900 K during the TPD measurement the work function was found to be 0.5 lower as compared to the value prior to the CO${}_{2}$ exposure. This is argued to be caused by a change in the surface morphology of the oxidized Sm/Ni system as a result of annealing.

### 3.3. Thermal Desorption Measurements

**Figure 6:**
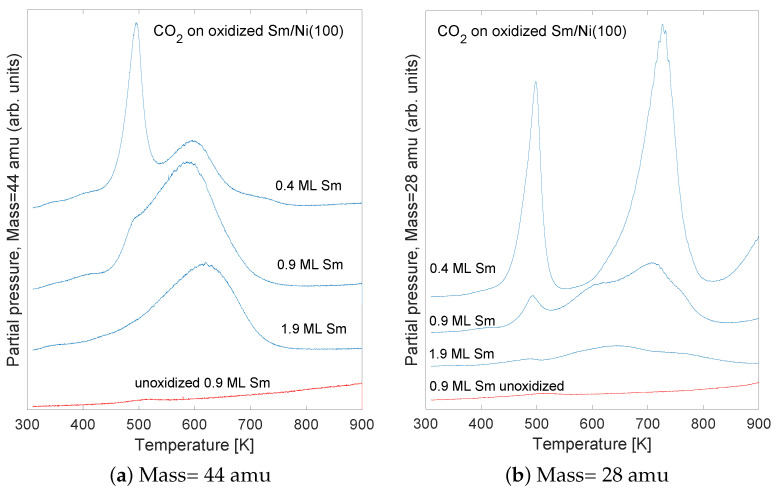
TPD spectra for 1L CO${}_{2}$ adsorbed on oxidized (top 3 spectra) and pure non-oxidized (bottom spectrum) Sm/Ni(100). The effective Sm coverage is indicated for each spectrum.

Carbon dioxide (mass 44 amu) TPD spectra are shown in Figure 6a for effective Sm coverage of about 0.4, 0.9, and 1.9 ML. Two distinct desorption features are observed for sub mono-layer Sm coverage; one near a temperature of 500 K and the other near 600 K. For the highest Sm coverage of 1.9 ML only the 600 K desorption peak was resolved. It seems likely that the Sm-Ni interface play a more important role in the case of sub-mono-layer Sm coverage as compared to multilayer coverage. It is therefore argued that the sharp TPD peak observed near 500 K for the 0.4 ML Sm coverage (top curve of Figure 6a) originates from the interface between O, Sm, and Ni. This peak becomes weaker for 0.9 ML Sm coverage, and has disappeared in the spectrum from the 1.9 ML Sm sample, where most of the surface is expected to be covered by Sm and CO${}_{2}$ is likely to adsorb on the Sm–O interface.

An interesting result in the present study is the observation of the difference in the desorption spectra when CO${}_{2}$ was exposed to pure non-oxidized Sm/Ni, and exposed to Sm/Ni that was exposed to oxygen. The effective Sm coverage was estimated to near 0.9 ML for both spectra. Very little CO${}_{2}$ was observed to desorb from the pure non-oxidized sample (bottom spectrum, Figure 6a). Similar results were found on samples of Sm coverage of 0.4 and 1.8 ML.

TPD spectra are shown in Figure 6b for mass 28 amu. These spectra were recorded simultaneously as the mass 44 amu spectra in Figure 6a. The peaks near temperatures 500 and 600 K are due to fragmentation of CO${}_{2}$ in the mass spectrometer, and the peak near 700 K most probably is due to desorption of carbon monoxide. The mass 28 amu fragment of CO${}_{2}$ has an intensity of about 25% of the main peak at 44 amu. The fragmentation pattern was measured by monitoring the relevant mass-spectrometer peaks when filling the vacuum chamber by CO${}_{2}$. From the TPD spectra of mass 28 and 44 amu it seems that most of the C adsorbed on the sample desorbs as CO${}_{2}$ upon heating. A smaller but significant fraction of surface carbon seem to desorb as carbon monoxide.

**Figure 7:**
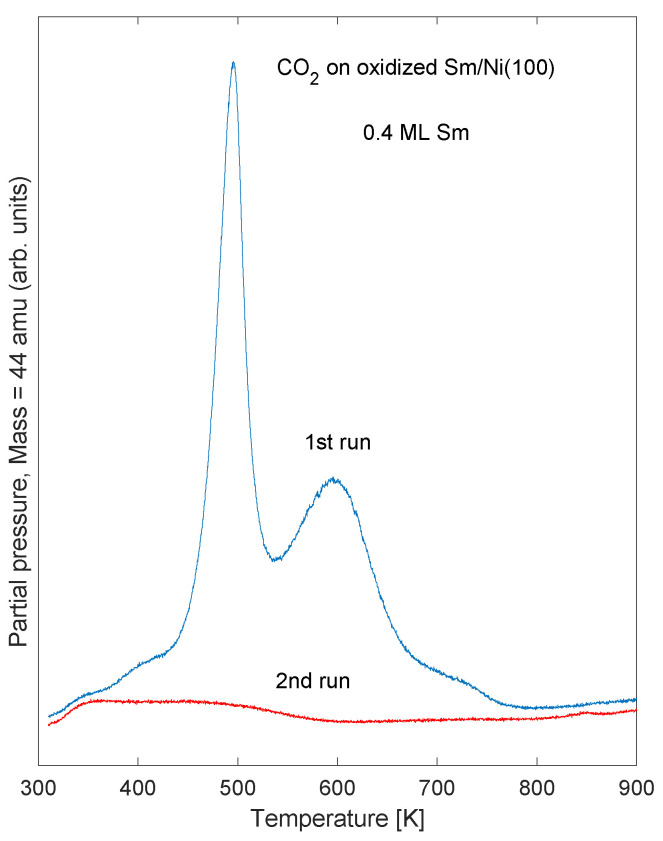
TPD spectra showing desorption of CO${}_{2}$ before (1st run) and after (2nd run) cycling the sample temperature to 900 K, for an oxidized sample of effective Sm coverage 0.4 ML.

Another observation was that very little carbon dioxide was adsorbed on the sample after temperature cycling up to 900 K and subsequently down to near 300 K. This is shown in Figure 7 for Sm coverage 0.4 ML. Similar observations were also seen for Sm coverage 0.9 and 1.9 ML. This finding is likely to be caused by changes in sample morphology upon heating, as also indicated by the XPS results.

## 4. Discussion

As mentioned in the introduction, CO${}_{2}$ tend to physisorb on metallic surfaces at low temperature and may chemisorb to form stronger bonds on oxide surfaces. The latter is the case for the oxidized Sm/Ni system as shown by desorption features in the temperature range from 450 to 700 K. This may be caused by an additional oxygen atom from the substrate which facilitates formation of carbonate species, as indicated by the carbon 1s core level spectra. Different adsorption pathways have been discussed in the review by Burghaus [1]. The simplest pathway may be described as

$${\mathrm{CO}}_{2}\left(gas\right)+O_{sur}\overset{}{\to }{\left[{\mathrm{CO}}_{2}\left(ads\right)-O_{sur}\right]}^{\#}\overset{}{\to }{\mathrm{CO}}_{3}\left(chem\right)$$

Another possible pathway may be that a bent ${\mathrm{CO}}_{2}^{\gamma -}species$ (carboxylate) acts as a precursor for CO${}_{2}$ adsorption

$${\mathrm{CO}}_{2}\left(gas\right)+O_{sur}\overset{}{\to }{\mathrm{CO}}_{2}^{\gamma -}+O_{sur}\overset{}{\to }{\left[{\mathrm{CO}}_{2}\left(ads\right)-O_{sur}\right]}^{\#}\overset{}{\to }{\mathrm{CO}}_{3}\left(chem\right)$$

where $O_{sur}$ indicates a oxygen site at the surface, # indicate an intermediate state, and gas, ads, and chem indicate gas phase, adsorbed species, and chemisorbed species, respectively.

Carbon dioxide is a non-polar linear molecule in which the bonds are polar. This gives a partial positive charge located on the carbon site and partial negative charges located on the oxygen sites. The 16 valence electrons are distributed among 8 molecular orbitals formed by linear combinations of atomic orbitals [31,32,33]. Bonding orbitals are $3\;\sigma_{g}$, $2\;\sigma_{u}$, and $1\;\pi_{u}$. The orbitals $4\;\sigma_{g}$, $3\;\sigma_{u}$, and $1\;\pi_{g}$ are occupied and non-bonding. The highest occupied molecular orbital (HOMO) is $1\;\pi_{g}$, and the lowest unoccupied molecular orbital (LUMO) is $2\;\pi_{u}$ [31,34,35,36,37]. In CO${}_{2}$ the LUMO is relatively low in energy, which facilitates charge transfer into the orbital for a molecule in the proximity of a surface.

**Figure 8:**
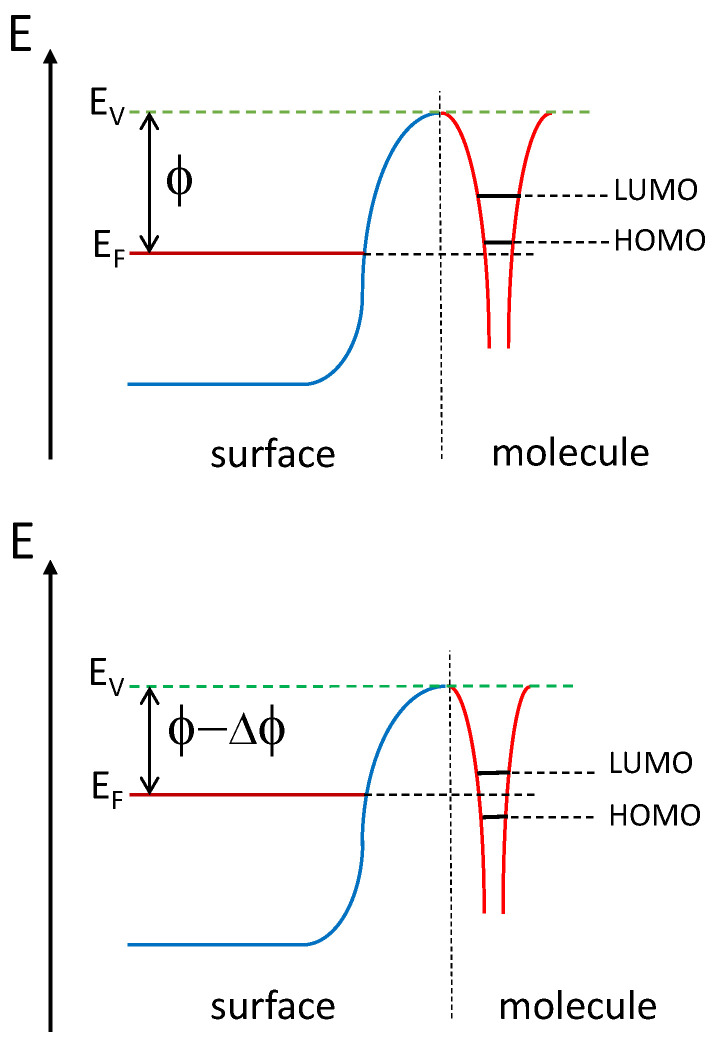
Schematic representation of relative changes in electron energy levels that result from a reduction in sample work function. The Fermi level and vacuum level are denoted $E_{F}$ and $E_{V}$, respectively. ϕ is the work function.

The finding that carbon dioxide is not adsorbed on the system after temperature cycling to 900 K (Figure 7) may be related to a reorganized surface structure as the sample is heated. It has previously been reported that thin rare earth overlayers tend to intermix with transition metal substrates and form surface alloys at relatively low temperatures [38]. The surface reactivity may be altered even if the observed changes in XPS intensities are small. The changed surface reactivity may be related to a decrease in work function of 0.5 eV (Table 2), and corresponding changes in energy differences between molecular levels and substrate Fermi level. Since the energy levels of the adsorbed molecule are referenced to the vacuum level, the value of the work function determines the energy difference between molecular orbitals and substrate Fermi level [39,40]. This is shown schematically in Figure 8. Therefore the probability of charge transfer between adsorbed molecule and substrate depends on the work function. The illustrated energy levels in the schematic figure do not represent true values. The decrease in work function by 0.5 eV as the Sm/Ni sample is heated to 900 K is likely to contribute to a change of the adsorption properties of CO${}_{2}$ of this sample. However, the details of the adsorption mechanisms of CO${}_{2}$ on oxidized Sm/Ni remain undetermined.

The core level intensities presented in Table 1 indicate changes in sample morphology upon heating. For the lowest Sm coverage of 0.4 and 0.9 ML a decrease in relative Sm and O intensities may suggest aggregation of Sm oxide structures on the Ni substrate [41]. For the largest Sm coverage, 1.9 ML, increased relative Sm and O intensities do not suggest aggregation, but still suggest structural changes in the surface nano structures, as does the change in work function.

Two peaks observed in the O 1s core level (Figure 3) are located at binding energies that are usually assigned to oxide (529–530 eV) and hydroxide (531 eV). In this case, hydroxide is not likely to be present and the high binding energy feature is speculated to be due to a mixed Sm–Ni oxide at the perimeter of the Sm nano structures. The shift in the low binding energy peak by 0.7 eV after heating is comparable to the change in the work function of 0.5 eV. The high binding energy peak does not shift after annealing, and is therefore speculated to originate from atoms in metallic contact with the Ni substrate, presumably in form of a mixed Sm–Ni oxide. The Sm 3d peaks (Figure 4) show a binding energy shift of 0.6 eV after annealing. Similar core level shifts that track changes in the work function have previously been observed on systems of thin oxide layers on conducting substrates, and have been argued to be caused by an inability to equilibrate Fermi levels on the short time scale of the photoemission process [42].

It should be noted that the amount of carbonate on the Sm/Ni sample that is exposed to oxygen is only on the order of a few percent on the surface. The role of carbonate is assumed to be crucial since no distinct CO${}_{2}$ desorption is observed in the absence of carbonate species, as witnessed by the C 1s XPS peak at binding energy near 290 eV. The TPD technique is sensitive enough to yield distinct desorption data for low desorption pressures. The low level (few percent) of carbon contamination on the Ni substrate is not likely to play a determining role for the observed desorption spectra from the O/Sm/Ni samples.

Future studies including measurements of surface structure are needed to obtain improved understanding of these phenomena. Studies of other supported rare earth systems will be of interest to this subject matter.

## Figures and Tables

**Table 1:** XPS intensities $I_{Sm}/I_{Ni}$ and $I_{O}/I_{Ni}$ of Sm 3d and O 1s relative to Ni 2p core levels for samples of effective Sm coverage of 0.4, 0.9, and 1.9 ML, and oxygen dose of 10 L. The intensities were corrected for atomic sensitivity factors. The label 1st run indicates measurements recorded before temperature ramp to 900 K, and 2nd run indicates measurements recorded after temperature ramp. ΔNi and ΔO show percentage change in relative intensities $I_{Sm}/I_{Ni}$ and $I_{O}/I_{Ni}$ after the temperature ramp.

Sample		$I_{\mathrm{Sm}}/I_{\mathrm{Ni}}$	$I_{O}/I_{\mathrm{Ni}}$	ΔSm	ΔO
0.4 ML Sm	1st run	0.10	0.17	-	-
	2nd run	0.09	0.06	−10%	−53%
0.9 ML Sm	1st run	0.24	0.28	-	-
	2nd run	0.22	0.18	−8%	−36%
1.9 ML Sm	1st run	0.71	0.70	-	-
	2nd run	0.73	0.76	+3%	+9%

**Table 2:** Work function values relative to Ni(100) for the following samples: (1) sample of effective Sm coverage of 0.8 ML on Ni(100); (2) sample 1 oxidized by 10 L $O_{2}$ at a temperature of 300 K; (3) sample 2 exposed to 1 L CO${}_{2}$ at 130 K; (4) sample 3 after annealing to 900 K. The work function of Ni(100) was measured to be 5.2 eV.

#	Sample	Work Function
	(Ni(100) Substrate)	[eV]
1	0.8 ML Sm/Ni	−2.3
2	Oxidized Sm/Ni	−1.2
3	CO${}_{2}$ on oxidized Sm/Ni	−0.6
4	after annealing	−1.7

## Data Availability

Experimental data obtained in this study may be obtained from the author.

## References

[1] Burghaus U. (2014). Surface chemistry of CO_2_. Adsorption of carbon dioxide on clean surfaces at ultrahigh vacuum. Prog. Surf. Sci..

[2] Rosynek M.P. (1977). Catalytic properties of rare earth oxides. Catal. Rev..

[3] Mullins D.R. (2015). The surface chemistry of cerium oxide. Surf. Sci. Rep..

[4] Cavani F., Trifirò F. (1999). Selective oxidation of light alkanes: Interaction between the catalyst and the gas phase on different classes of catalytic materials. Catal. Today.

[5] Yuan K., Zhang Y.W. (2020). Engineering well-defined rare earth oxide-based nanostructures for catalyzing C1 chemical reactions. Inorg. Chem. Front..

[6] Falicov L.M., Somorjai G.A. (1985). Correlation between catalytic activity and bonding and coordination number of atoms and molecules on transition metal surfaces: Theory and experimental evidence. Proc. Natl. Acad. Sci. USA.

[7] Külah E., Marot L., Steiner R., Romanyuk A., Jung T.A., Wäckerlin A., Meyer E. (2017). Surface chemistry of rare-earth oxide surfaces at ambient conditions: Reactions with water and hydrocarbons. Sci. Rep..

[8] Lundy R., Byrne C., Bogan J., Nolan K., Collins M.N., Dalton E., Enright R. (2017). Exploring the Role of Adsorption and Surface State on the Hydrophobicity of Rare Earth Oxides. ACS Appl. Mater. Interfaces.

[9] Carchini G., García-Melchor M., Lodziana Z., Lopez N. (2016). Understanding and Tuning the Intrinsic Hydrophobicity of Rare-Earth Oxides: A DFT+U Study. ACS Appl. Mater. Interfaces.

[10] Lawrence J.M., Riseborough P.S., Parks R.D. (1981). Valence fluctuation phenomena. Rep. Prog. Phys..

[11] Wertheim G.K., Crecelius G. (1978). Divalent Surface State on Metallic Samarium. Phys. Rev. Lett..

[12] Raaen S., Parks R.D. (1983). Surface versus shake-down effects in the deep core photoemission of Sm-based and Eu-based intermetallics. Phys. Rev. B.

[13] Adachi G., Imanaka N. (1998). The binary rare earth oxides. Chem. Rev..

[14] Dri C., Peronio A., Vesselli E., Africh C., Rizzi M., Baldereschi A., Peressi M., Comelli G. (2010). Imaging and characterization of activated CO_2_ species on Ni(110). Phys. Rev. B.

[15] Ding X., Rogatis L.D., Vesselli E., Baraldi A., Comelli G., Rosei R., Savio L., Vattuone L., Rocca M., Fornasiero P. (2007). Interaction of carbon dioxide with Ni(110): A combined experimental and theoretical study. Phys. Rev. B.

[16] Vesselli E., Rizzi M., Rogatis L.D., Ding X., Baraldi A., Comelli G., Savio L., Vattuone L., Rocca M., Fornasiero P. (2010). Hydrogen-Assisted Transformation of CO_2_ on Nickel: The Role of Formate and Carbon Monoxide. J. Phys. Chem. Lett..

[17] Wang S.G., Cao D.B., Li Y.W., Wang J., Jiao H. (2005). Chemisorption of CO_2_ on Nickel Surfaces. J. Phys. Chem. B.

[18] Vesselli E., Rogatis L.D., Ding X., Baraldi A., Savio L., Vattuone L., Rocca M., Fornasiero P., Peressi M., Baldereschi A. (2008). Carbon Dioxide Hydrogenation on Ni(110). J. Am. Chem. Soc..

[19] Roiaz M., Monachino E., Dri C., Greiner M., Knop-Gericke A., Schlög R., Comelli G., Vesselli E. (2016). Reverse Water—Gas Shift or Sabatier Methanation on Ni(110)? Stable Surface Species at Near-Ambient Pressure. J. Am. Chem. Soc..

[20] Vesselli E., Schweicher J., Bundhoo A., Frennet A., Kruse N. (2011). Catalytic CO_2_ Hydrogenation on Nickel: Novel Insight by Chemical Transient Kinetics. J. Phys. Chem. C.

[21] Kattel S., Ramírez P.J., Chen J.G., Rodriguez J.A., Liu P. (2017). Active sites for CO_2_ hydrogenation to methanol on Cu/ZnO catalysts. Science.

[22] Gokhale A.A., Dumesic J.A., Mavrikakis M. (2007). On the Mechanism of Low-Temperature Water Gas Shift Reaction on Copper. J. Am. Chem. Soc..

[23] Skovbjerg L.L., Okhrimenko D.V., Khoo J., Dalby K.N., Hassenkam T., Makovicky E., Stipp S.L.S. (2013). Preferential Adsorption of Hydrocarbons to Nanometer-Sized Clay on Chalk Particle Surfaces. Energy Fuels.

[24] Stoch J., Gablankowska-Kukucz J. (1991). The Effect of Carbonate Contaminations on the XPS 0 1s Band Structure in Metal Oxides. Surf. Interface Anal..

[25] Campagna M., Wertheim G.K., Baer Y., Ley L., Cardona M. (1979). Unfilled inner shells: Rare earths and their compounds. Photoemission in Solids II.

[26] Utsumi Y., Kasinathan D., Ko K.T., Agrestini S., Haverkort M.W., Wirth S., Wu Y.H., Tsuei K.D., Kim D.J., Fisk Z. (2017). Bulk and surface electronic properties of *SmB*_6_: A hard x-ray photoelectron spectroscopy study. Phys. Rev. B.

[27] Suzuki C., Kawai J., Takahashi M., Vlaicu A.M., Adachi H., Mukoyama T. (2000). The electronic structure of rare-earth oxides in the creation of the core hole. Chem. Phys..

[28] Kuntaiah K., Sudarsanam P., Reddy B.M., Vinu A. (2013). Nanocrystalline *Ce*_1−x_*Sm*_x_*O*_2−d_(*x* = 0.4) solid solutions: Structural characterization versus CO oxidation. RSC Adv..

[29] Thomas S., Sherwood P.M.A. (1993). Valence Band X-ray Photoelectron Spectroscopic Studies of Carbonate, Bicarbonate and Formate Interpreted by X*α* Calculations. Surf. Interface Anal..

[30] Sherwood P.A. (1991). Carbon, functionalized carbon, and hydrocarbons studied by valence band photoemission. J. Vac. Sci. Technol. A.

[31] Paparo A., Okuda J. (2017). Carbon dioxide complexes: Bonding modes and synthetic methods. Coord. Chem. Rev..

[32] Freund H.J., Roberts M.W. (1996). Surface chemistry of carbon dioxide. Surf. Sci. Rep..

[33] Taifan W., Boily J.F., Baltrusaitis J. (2016). Surface chemistry of carbon dioxide revisited. Surf. Sci. Rep..

[34] Allan C.J., Gelius U., Allison D.A., Johansson G., Siegbahn H., Siegbahn K. (1972). ESCA studies of CO_2_, CS_2_ and COS. J. Electron Spectrosc. Relat. Phenom..

[35] Pacansky J., Wahlgren U., Bagus P.S. (1975). SCF *ab-initio* ground state energy surfaces for CO_2_ and CO_2_^−^. J. Chem. Phys..

[36] Aresta M., Angelini A., Lu X.B. (2016). The carbon dioxide molecule and the effects of its interaction with electrophiles and nucleophiles. Carbon Dioxide and Organometallics.

[37] Johnson P.M., Albrecht A.C. (1966). Carbon dioxide as an electron trap in 3-Methylpentane at 77 K. J. Chem. Phys..

[38] Braaten N.A., Grepstad J.K., Raaen S. (1989). Photoemission study of formation and oxidation of a cerium-copper interface. Phys. Rev. B.

[39] Raaen S., Hunvik K.W.B. (2020). Non-activated adsorption of methane on nickel surfaces induced by reduced work function. Appl. Surf. Sci..

[40] Raaen S., Braaten N.A. (1990). Referencing core levels in photoelectron spectroscopy. Phys. Rev. B.

[41] Hunvik K.W.B., Støvneng A., Pacáková B., Raaen S. (2019). CO desorption from nickel-decorated muscovite mica. Appl. Surf. Sci..

[42] Raaen S. (1991). Correspondence between the work function and overlayer core level shifts in oxidized cesium on carbon. Phys. Rev. B.

